# REPAC: analysis of alternative polyadenylation from RNA-sequencing data

**DOI:** 10.1186/s13059-023-02865-5

**Published:** 2023-02-09

**Authors:** Eddie L. Imada, Christopher Wilks, Ben Langmead, Luigi Marchionni

**Affiliations:** 1 Department of Pathology and Laboratory Medicine, Weill Cornell Medicine, New York, USA; 2 Department of Computer Science, Johns Hopkins University, Baltimore, USA

**Keywords:** Polyadenylation, Method, Compositions

## Abstract

Alternative polyadenylation (APA) is an important post-transcriptional mechanism that has major implications in biological processes and diseases. Although specialized sequencing methods for polyadenylation exist, availability of these data are limited compared to RNA-sequencing data. We developed REPAC, a framework for the analysis of APA from RNA-sequencing data. Using REPAC, we investigate the landscape of APA caused by activation of B cells. We also show that REPAC is faster than alternative methods by at least 7-fold and that it scales well to hundreds of samples. Overall, the REPAC method offers an accurate, easy, and convenient solution for the exploration of APA.

**Supplementary Information:**

The online version contains supplementary material available at 10.1186/s13059-023-02865-5.

## Background

Mechanisms that control gene expression at the RNA level are often referred to as post-transcriptional regulation (PTR) mechanisms. Splicing and alternative polyadenylation (APA) are well-known examples of PTR that can regulate not only gene expression but also their function. While splicing has been extensively studied since the advent of next generation sequencing (NGS), APA studies are far less common than splicing studies. Indeed, inferring APA events from RNA-Seq data is challenging due to the lack of an intrinsic characteristic (e.g., split-reads for splicing), and for this reason, several specialized sequencing methods were developed to pinpoint polyadenylation sites (PAS) [1–3]. Although these methods improve the quantification of PA sites usage, the number of publicly available data derived from these methods is extremely limited in comparison to traditional RNA-Seq data.

This poses a challenge to the study of polyadenylation (PA) biology given that the lack of publicly available data severely hinders investigation and hypothesis generation without incurring major experimental costs. To overcome this limitation, several groups have developed methods to quantify PA usage from RNA-Seq data [4–6]. Among them, DaPars [6] is a popular method to compare PA profiles across two phenotypes and detect the differential usage of APA by the degree of difference in APA usage quantified as a change in percentage of distal polyA site usage Index ($\Delta PDUI$). Likewise, QAPA [4], which leverages the speed and power of pseudo-alignment software, such as salmon, was shown to be the fastest method for APA studies so far.

Most of the methods currently available rely on the effect size of the change in the proportion of the expression levels between different PA sites. Performing statistical analysis on proportions of a total (i.e. percentage) imposes many statistical limitations that are often ignored and can lead to inaccurate results. Most multivariate methods that were developed for real values cannot be directly applied to compositional data (proportional data) because compositional data often breaks many assumptions of these methods [7]. Moreover, these methods do not allow for the control of unwanted variables or the design of more complex comparisons (e.g., factorial designs, paired samples, etc.).

In this work, we present regression of polyadenylation compositions (REPAC) a novel framework to detect differential alternative polyadenylation (APA) using regression of polyadenylation compositions which can appropriately handle the compositional nature of this type of data while allowing for complex designs. We show that REPAC is faster and yields more accurate and robust results in comparison to other methods.

## Results

### REPAC can accurately detect APA events

**Fig. 1:**
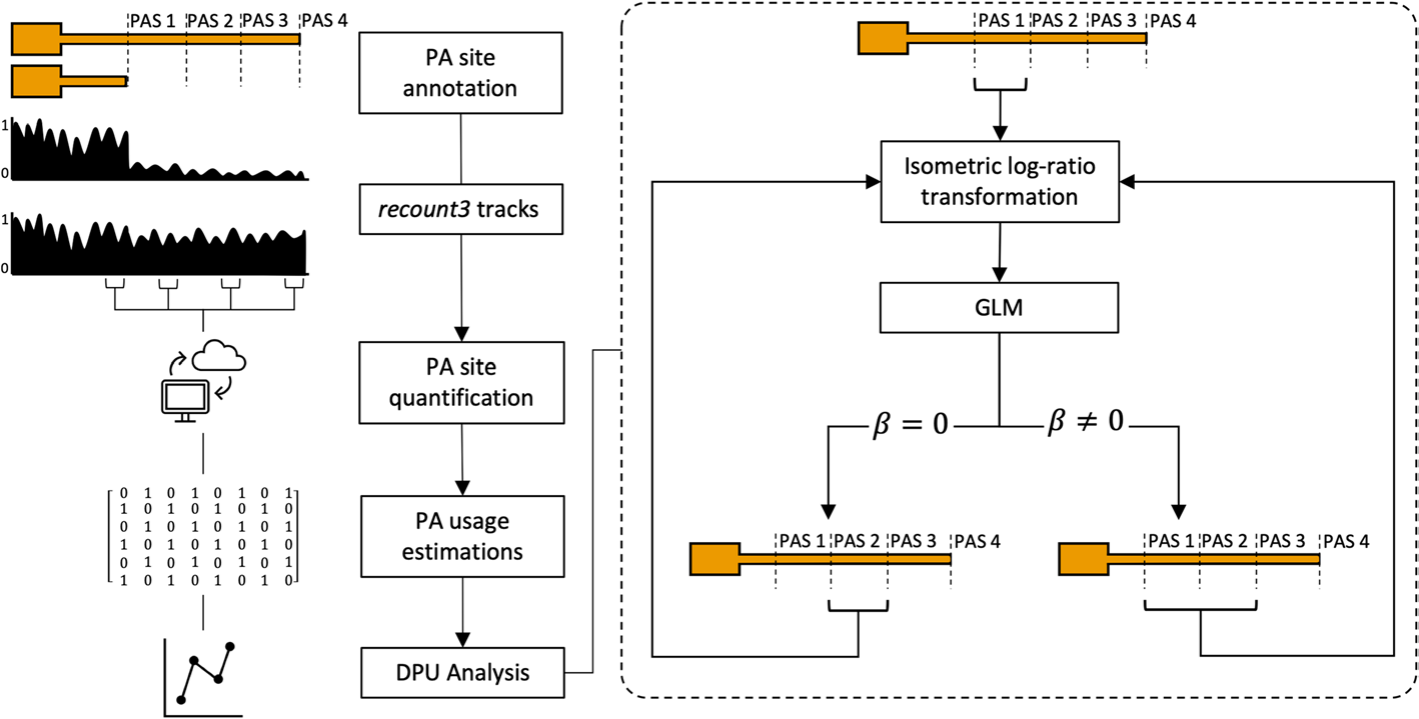
REPAC workflow. REPAC perform analysis of differential polyadenylation usage by analyzing the upstream region of annotated PAS. While quantification of PAS can be performed in traditional ways (alignment and counting), it was primarily design to take advantage of the *recount3* project to extract counts on-the-fly for over 750,000 samples publicly available

**Fig. 2:**
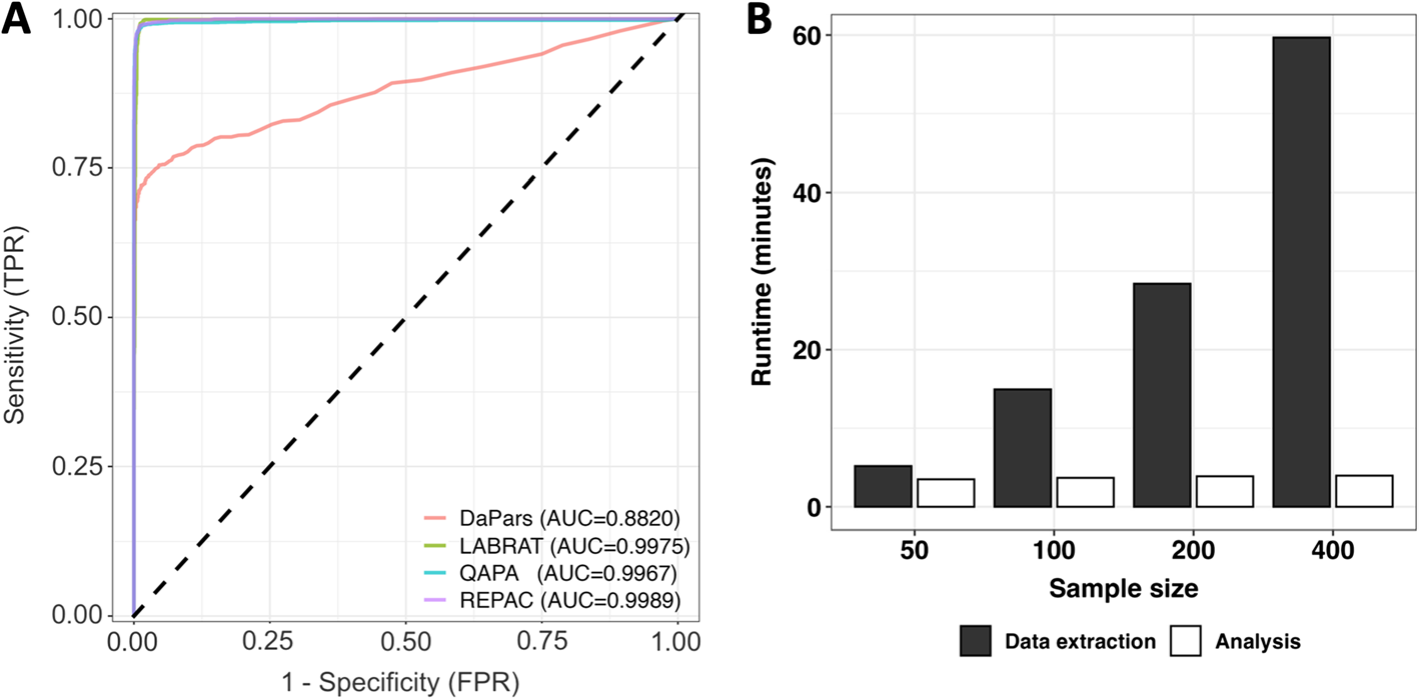
Benchmarked tests. **A** Empirical ROC curves of REPAC, QAPA, and DaPars. REPAC and QAPA performs very similarly ($AUC = 0.99$) in simulated data, while DaPars shows a lower performance ($AUC=0.88$). Curves were based on the effect-size of the methods i.e., cFC, $\Delta PDUI$ and $\Delta PAU$ for REPAC, DaPars, and QAPA, respectively. **B** REPAC runtime scales almost linearly with the number of samples

REPAC makes use of expression estimates of 50 bp window upstream of annotated PA sites to fit a generalized linear regression model on the compositions to assess differential polyadenylation site usage (DPU) occurring between conditions. This quantification can be done with traditional tools (e.g., Subread, HT-Seq, etc.) or directly pulled from *recount3 *[8] bigWig files (Fig. 1). Briefly, to assess the performance of REPAC, we simulated 2 conditions (*n *= 5 for each condition) with 5000 genes having a longer or shorter isoform. Half of the set had one of the isoforms predominantly expressed in one of the conditions (1250:1250 for short/longer isoforms), and the other half did not have a predominant isoform. The ratio of longer to shorter isoforms was randomly defined within a pre-defined range (see the “Methods”). We applied the REPAC method after removing 197 low expressed genes. For each type of event, REPAC was able to achieve 0.99/0.98, 0.99/0.98 and 0.98/0.98 specificity/sensitivity for lengthening, shortening, and no preference (NP), respectively. With an overall accuracy of 0.98 and an area under the curve (AUC) of 0.9989, REPAC was shown to accurately detect APA events (Fig. 2A).

### Performance of alternative methods for APA detection

We compared the performance of REPAC to other popular methods for APA detection: DaPars [6], QAPA [4], and LABRAT [9]. Both QAPA and LABRAT relies on external tools such as salmon to obtain estimates of each isoform 3′-UTR expression level which are transformed to relative expression levels by normalizing the isoform expression level by the total 3’-UTR expression level. However, LABRAT implements significance testing for the differences in PAS usage, which QAPA does not. In contrast, DaPars relies on 3′-UTR changes in coverage to infer de novo PA sites and their differential usage.

We measured the performance of all the methods using the same simulation experiment. For each method, we used their respective effect size estimators ($\Delta PDUI$ for DaPars and $\Delta PPAU$ for QAPA) to compute the AUC similarly to what done for REPAC. Out of the four methods tested, REPAC, QAPA, and LABRAT were able to achieve high accuracy, with REPAC presenting a marginally higher performance (AUC = 0.9989 versus 0.9967 and 0.9975 for QAPA and LABRAT, respectively), while DaPars exhibited the lowest performance in the simulated dataset (AUC = 0.882) (Fig. 2A). The lower performance of DaPars was on par with previous simulations suggesting that de novo prediction of PAS from traditional RNA-seq data is relatively inaccurate compared to methods that rely on annotated PAS [4]. Moreover, DaPars was drastically slower (> 40-fold) than all the methods tested (Additional file 1: Fig. S1).

### REPAC enables fast and streamlined exploration of public domain data

The REPAC method and associated R package were designed to take advantage of our recently published recount3 resource [8]. The recount3 project processed and analyzed data from over 750,000 samples of human and mouse origin publicly available in SRA in a standardized manner. The REPAC package extends recount3 to enable the extraction of expression estimates for thousands of PA sites within a few seconds per sample. The REPAC framework has major advantages over existing methods for APA analysis from RNA-seq data. For instance, it allows the user to skip the time- and storage-intensive step of downloading the raw data and processing it. By integrating with the recount3 framework, REPAC enables researchers to explore differential APA events for thousands of phenotypes in a fast and streamlined fashion. Since the protocols used to generate publicly available data sets can vary greatly, we performed three additional simulations to assess the performance of REPAC on common protocol variations. These variations were as follows: (1) polyA capture vs ribosomal depletion, (2) 50bp vs 75bp reads, (3) single- vs paired-end reads, and (4) experiment with factorial design. Overall, we found that REPAC is robust to variations in protocols with an overall accuracy greater than 0.95 in all simulations (see full benchmark statistics in Additional file 2: Table S1). QAPA and LABRAT both relies on lightweight quasi-mapping implemented in the salmon software, which has already been shown to be substantially faster than other methods such as DaPars and ROAR. Therefore, we benchmarked the speed of REPAC against QAPA and LABRAT. To this end, we started by obtaining from SRA the raw data for the project SRP048707 (see below). The total size of the data set analyzed was approximately 27 GB (gzipped fastq files). Assuming a constant speed of 300 Mbps, this step alone would take approximately 15 min to complete. Because REPAC pulls expression estimates directly from recount3, it allows the users to skip this step. Next, pre-processing (extracting 3′-UTR sequences, building the index and quantification) and expression quantification with salmon [10] for QAPA analysis took on average 17 min and LABRAT took on average 20 min. Meanwhile, obtaining PAS estimates with the REPAC package took under one minute. Finally, detecting DPU between the comparisons took 0.03 min with QAPA and 6.5 min with LABRAT, compared to 3.2 min with REPAC. However, it is important to note QAPA does not perform any statistical testing, which contributes to the speed in this step. Overall, considering the entire workflow of an APA study, REPAC was 7.6 times faster than QAPA and 8.3 times faster than LABRAT.

### The REPAC method is scalable to large data sets

One advantage of REPAC is its ability to tap into recount3 to directly obtain PAS expression quantification. As demonstrated in the previous analysis, this feature can greatly speed up the analytical process even for small data sets. However, the advantages of REPAC become abundantly clear when analyzing large collections, such as the GTEx for instance, for which storage and computing power requirements can quickly become a limiting factor for many researchers. Moreover, the process of acquiring access to raw data can be slow and burdensome. To test how well REPAC scales with increasing amounts of data, we extracted PAS quantification and performed a DPU analysis for a total of 20, 100, 200, and 400 randomly selected brain (cortex) and testis tissue samples from the GTEx project (V8). We found that the time to quantify PAS with REPAC scaled linearly with the number of samples, with an average of 9 seconds per sample. The time to test for DPU between conditions remained stable at around four minutes (Fig. 2B).

When looking at highly differential PA site usage ($|cFC| \ge 0.25$, $FDR \le 0.05$) between 200 testis and 200 brain samples, we observed 879 genes with differential usage of PAS. The results showed that testis favors the expression of shorter 3’-UTR isoforms when compared to the cerebral cortex, with over 97% of the genes detected showing preferential usage of a shorter PAS in testis and vice-versa. These results were consistent with previous studies reports [11, 12] (Additional file 1: Fig. S2).

### Global profiling of APA during B cell activation

**Fig. 3:**
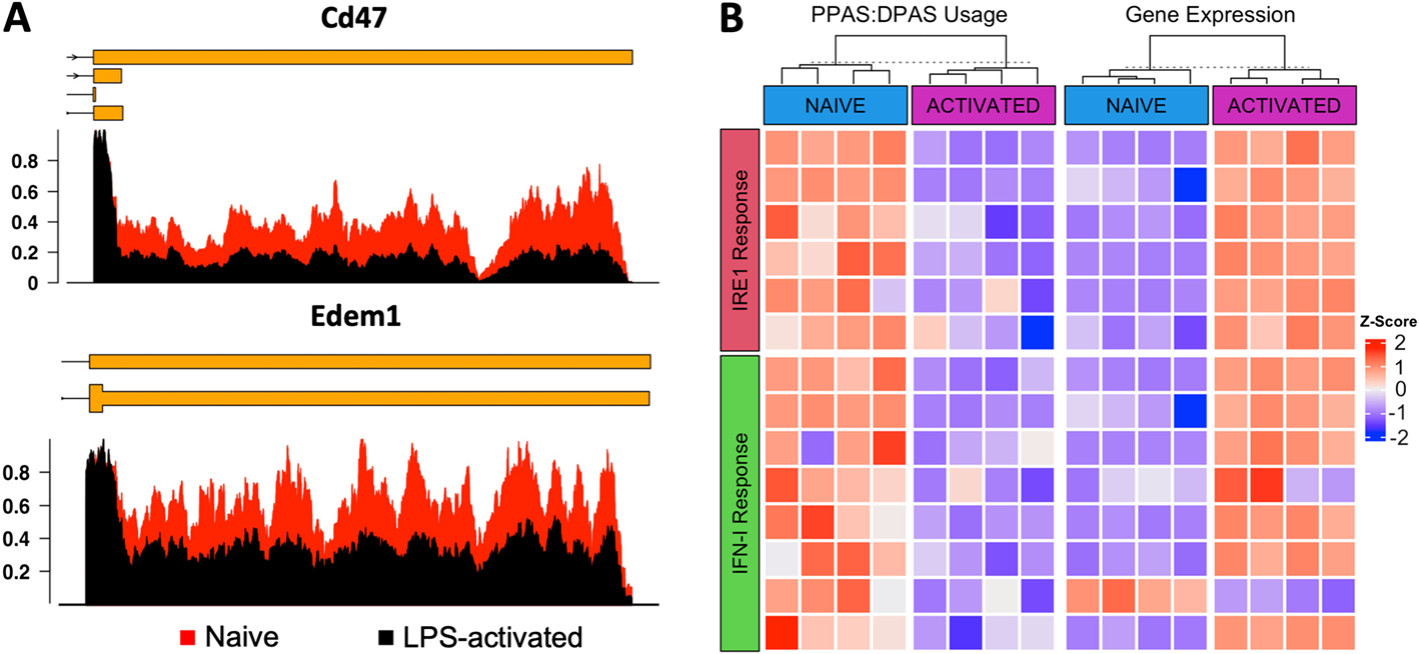
3′-UTR-shortening events in naive and LPS-activated B cells. **A** Differences in 3′-UTR coverage of secretion associated genes Cd47 and Edem1. The coverage was normalized by the highest expressing base pair. **B** Heatmap showing the relationship between the usage of the proximal and distal PAS (PPAS:DPAS) and expression levels (CPM) using standardized values. Overall, preferential usage of the shorter 3′-UTR isoform (blue color) is associated with higher expression levels (red color) for the genes involved in secretion and IFN-I response

We applied the REPAC method to investigate the landscape of APA in response to B cell activation. To this end, we used a dataset from Diaz-Muñoz et al. [13] containing naive and LPS-activated B cells (with four replicates per condition). Using the REPAC package, we obtained expression estimates for 67,509 3′-UTR PAS (as derived from the PolyASite 2.0 database [14], see the “Methods”) for all samples and then performed a DPU analysis comparing naive versus LPS-activated B cells. This analysis detected 117 genes with DPU in response to B cell activation ($|cFC| \ge 0.20$, adjusted *p*-value $\le 0.01$). Approximately 80% of the genes with DPU were found to have a higher usage of a more proximal PAS upon B cell activation, with the 3′-UTR being 817 bp shorter on the median. We found that genes associated with secretion mechanisms, such as Cd47, Edem1, and Rbx1, were among the most significant DPU events (Fig. 3A). Next, we investigated whether these changes were associated with a particular biological process (BP). Through gene set enrichment analysis (GSEA) of GO BP, we observed that 19 BP were significantly enriched in 3′-UTR-Shortening (3′-US) events (adjusted *p*-value$\le 0.05$). Among the top enriched pathways were IRE1 mediated unfolded protein response and response to type I interferon (IFN-I) (Fig. 3B). Some of these findings were consistent with findings from Cheng and collaborators [15] showing that in activated B cells, the genes associated with secretion exhibit shorter 3′-UTRs. Notably, our analysis also expanded the currently known set of genes and processes affected by APA-revealing processes, such as response to IFN-I, that have not been associated with B cell activation before.

Additionally, we also performed the same comparison using QAPA and LABRAT to assess whether these methods could also recover known B cell biology. The analysis by QAPA was able to detect 28 genes with DPU ($\Delta |PPAU| \ge 20$), while LABRAT was able to detect 137 genes with DPU ($\psi \ge 0.1$, $FDR \le 0.01$). GSEA analysis on QAPA and LABRAT results did not indicate enrichment in any of the processes found enriched by REPAC, including secretion pathways previously reported by other studies [15] (see GSEA enrichment results in Additional file 2: Tables S2-S4).

Surprisingly, none of the APA events were detected in common by all methods. Given this discrepancy, we visually evaluated inspect the results for the top 10 predicted events. Upon visual inspection of the results, we found that the events predicted by QAPA were largely driven by false positives caused by low 3′-UTR coverage in one of the groups, and no striking changes were observed in genes with enough coverage for QAPA and LABRAT (see Additional file 1: Figs. S3 and S4), while the events predicted by REPAC clearly showed a difference in 3′-UTR coverage (see Additional file 1: Fig. S5).

### Technical implications of APA in downstream analyses

**Fig. 4:**
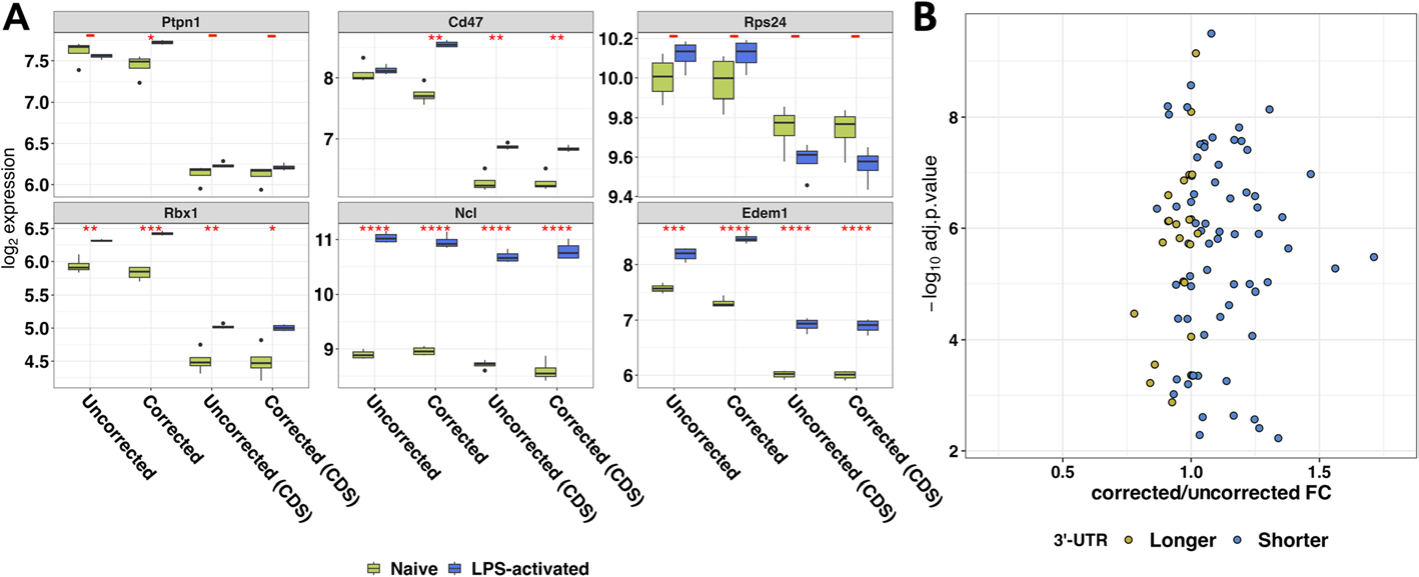
APA can drastically impact expression estimates. **A** Expression levels of the whole transcript and CDS-only with and without length correction by tximport for the top 6 APA events predicted by REPAC. Many genes can be found deferentially expressed after length correction, highlighting the importance of APA in other analysis. *P*-values from a *t*-test between groups are shown. **B** Scatter plot shows the ratio between fold-change estimated from a differential gene expression analysis with and without length correction

Our results indicated that APA events in 3′-UTR regions can drastically impact transcript size, as previously reported by other studies [9, 16]. Therefore, we investigated how APA events can impact downstream steps, such as differential gene expression analysis. To this end, we obtained length-corrected (length scaled TPM) and uncorrected gene expression estimates via the salmon-tximport pipeline from whole transcript and CDS-only sequences. Next, we carried out a differential gene expression analysis between naive and activated B cells. After taking into account the different lengths of the isoforms, we found that many genes with APA events captured by REPAC exhibited significant changes in expression levels and fold-change between conditions ($|logFC| \ge 0.5$, adjusted *p*-value $\le 0.05$; Fig. 4A).

This issue was evidenced by genes such as Cd47, Ptpn1, and Edem1, whose expression levels after LPS-activation increase by 75%, 222%, and 20%, respectively, when adjusting for the transcript length. In contrast, if the transcript length is not taken into account, the observed changes in expression level upon LPS-activation for Cd47, Ptpn1, and Edem1 are 2%, 151%, and − 4.7%, respectively (Fig. 4A). Moreover, for quantification of CDS regions, length-correction had little or no effect on gene expression levels of the majority of selected genes, confirming that the differences observed after correction in the whole transcript quantification did not originate from alternative splicing events. Genes predicted by REPAC to have 3′-US upon B cell activation showed predominantly increased fold-change values, while genes predicted to have 3′-UTR lengthening (3’UL) presented a decrease in fold-change when comparing fold-changes before and after isoform length correction (Fig. 4B). For some genes, we did not observe significant changes in fold-change after correction due to alternative PAS for these genes not being annotated as independent isoforms meaning these differences are usually not captured by gene expression quantification software.

## Discussion

Here, we present regression of polyadenylation compositions (REPAC), a new framework for the study of differential PA events using traditional bulk RNA-sequencing data. The REPAC method is based on the principles of compositional data (CD) analysis developed by Aitchson [7].

In our simulated data set REPAC outperformed DaPars, which is currently, one of the most popular methods of APA analysis, in both accuracy and speed. When compared to more recent developments such as QAPA, REPAC presented a marginal increase in performance (Fig. 2A). However, when comparing the results of REPAC, QAPA, and LABRAT in in a case-study, we found that REPAC was able to recover more known APA events associated with B cell activation than QAPA and LABRAT (Additional file 2: Tables S2-S4). Moreover, REPAC offers substantial advantages over other existing methods.

The REPAC package takes full advantage of the recount3 framework to skip the data acquisition and processing steps and directly extract the necessary data for differential APA analysis with little resource usage and time. This feature makes over 750,000 samples readily available for analysis (Fig. 1). This is a huge advantage over existing methods since all of them require raw data to be obtained and processed before analysis. Even when disregarding the time and effort of data acquisition, DaPars and QAPA still require pre-processing of the data such as aligning the data to the genome or performing 3′-UTR estimations with salmon, respectively. Despite salmon being relatively lightweight and faster than alignment-based methods (i.e. DaPars), it still requires substantial computational resources to carry the index construction and quantification, especially when a large number of PAS are used. Additionally, since REPAC makes use of generalized linear regression models, it can easily handle complex comparisons (i.e. factorial designs, paired samples, etc.) and correct for unwanted sources of variations (i.e. batch effects, blocks, etc.), which cannot be directly modeled by other methods (Additional file 2: Table S1).

All of these overheads translate into a much faster and more accurate analysis with REPAC than other methods. We demonstrated that the analysis of a small data set (*n *= 8) was 7.6 times faster to process with REPAC than QAPA (4.2 versus 32 min) and LABRAT (4.2 versus 35 min). Both of these methods already outperforms other methods[4, 9]. Even when disregarding the time for data acquisition, REPAC was still 4 times faster than QAPA and 4.8 times faster than LABRAT. It is important to note that these differences in processing times would significantly scale with larger data sets, to the point where performing APA analysis with other methods, might become infeasible for research groups without access to a robust computing environment.

As a proof of principle, we used REPAC to compare brain (cortex) and testis tissues from GTEx V8. On average, REPAC took 9 seconds per sample to quantify all PAS, meaning REPAC is scalable for large data sets with 400 samples taking under one hour to complete the entire analysis (Fig. 2B). In contrast, other methods would require the user to first get permission to access the raw data, download the data, and processes it, all of which would take days and access to a robust computing environment that are compliant with data privacy laws (i.e. Health Insurance Portability and Accountability Act in the US, and General Data Protection Regulation in Europe).

Moreover, the results of this comparison were consistent with previous studies reporting that cells from non-proliferative tissue (e.g., brain) tend to express longer 3′-UTR isoforms than cells from proliferative tissues (eg, testis) [11, 12]. Interestingly, we found that 3′-US events were enriched for genes involved in spermatogenesis and androgen response, suggesting that APA is not only associated with a proliferative state but also regulates specific processes associated with tissue function (Additional file 1: Fig. S2).

Despite one of the first pieces of evidence of APA as a functional mechanism being reported during B cell transition to plasma cell [17], the landscape of APA during B cell activation had remained largely under-explored. Recently, a study by Cheng and collaborators [15] conducted a broad survey of APA in secretory cell differentiation and observed that many genes involved in secretion presented 3′-UTR shortening after B cell activation. In light of these findings, we performed a case-study in order to evaluate if REPAC and other methods were able to replicate the findings by Chen and collaborators [15].

Our analysis of naive versus LPS-activated B cells detected hundreds of genes impacted by APA, whose majority were 3′-UTR shortening events. On par with previous observations[15], we found that genes involved in secretion were enriched in 3′-UTR shortening. Specifically, we found that genes involved in the IRE1 mediated unfolded protein response enriched in shortening events (Fig. 3B). We also observed that the genes involved in this pathway exhibited a negative correlation between 3′UTR size and gene expression, a relationship which has already been extensively reported by other studies [9, 16]. Surprisingly, QAPA was not able to capture APA across any gene involved in the secretion pathway. LABRAT managed to capture one gene involved in secretion (Edem11) but did not exhibited any enrichment in processes related to secretion.

Interestingly, response to type I interferon (IFN-I) was among the most enriched processes in 3′-US. Early response of B cells to IFN-I has been shown to elicit many types of responses (e.g., enhance antiviral humoral feedback by increasing the formation of early antiviral IgM, increase TLR-9 mediated activation and regulate autoreactive B cell activation [18–20]). Using REPAC, we were able to detect 3′-US in genes such as Ptpn1, Ube2k, and Irf2bp2, which are known to be involved in cell response to interferon stimulation [21–25].

Finally, we also demonstrated that APA has serious implications on downstream analysis (e.g., differential gene expression analysis) since traditionally gene length is assumed to be the same between conditions and therefore is not accounted for in many approaches. While recent pipelines based on expression estimates at transcript level through quasi-mapping, such as the salmon-tximport [10, 26] pipeline used in this analysis, can correct for differential isoform/3′-UTR usage, they are only able to do so if these isoforms/3′-UTRs are properly annotated. In this regard, we found that major annotation resources like GENCODE/ENSEMBL and RefSeq still lack proper annotation of 3’-UTRs for many genes. Therefore, the accessibility and ease of usage of REPAC is a powerful tool in making sure APA events are properly detected, annotated, and studied.

## Conclusions

We demonstrated that REPAC is a robust and powerful tool for exploring the biology of APA. It enables the analysis of over 750,000 samples encompassing thousands of different phenotypes. With REPAC, we want to encourage more studies on APA and how they influence normal and disease tissues. We hope this new tool can help pave the way to develop new hypotheses that can be further explored to understand the biological role of APA as a whole.

## Methods

### Detection of differential polyadenylation site usage

The REPAC framework is based on the Aitchison geometry in the simplex. A D-part simplex is defined as:

1 $$\begin{aligned} S^{D} = \left\{ {\textbf {x}}=\left[ x_{1}, x_{2}, \dots , x_{D} \right] ; x_{i}>0, i=1,2,\dots ,D; \sum _{i=1}^{D}x_{i}=k \right\} \end{aligned}$$

where *D* is the number of elements of comprising a composition (i.e., number of PAS) and *k* is a positive constant. We apply the isometric log ratio (*ilr*) transformation to the simplex. Let **x** be a D-part simplex, then:

2 $$\begin{aligned} clr({\textbf {x}})= & {} \left[ ln\frac{x_{1}}{g({\textbf {x}})}, ln\frac{x_{2}}{g({\textbf {x}})}, \dots ,ln\frac{x_{D}}{g({\textbf {x}})}\right] \nonumber \\ ilr({\textbf {x}})= & {} clr({\textbf {x}})U^t \end{aligned}$$

where $g({\textbf {x}}) = \left[ x_{1} x_{2} \dots x_{D} \right] ^\frac{1}{D}$ is the geometric mean of the composition and $U^t$ is a matrix which columns form an orthonormal basis of the centered log-ratio (*clr*) plane of **x**. Starting with a initial reference *r* (most proximal PAS, $r_{0}=x_{1}$), we iterate over each subsequent PAS ($t=[x_{2}, \dots , x_{D}]$) testing for DPU by fitting a linear model in a ilr-transformed sub-composition comprised of a reference and a target *C*[*r*, *t*], updating the reference to the target whenever the difference in compositions is not significant.

3 $$\begin{aligned} Y_n= & {} ilr\left( C \left[ x_{r}, x_{t} \right] \right) ,\nonumber \\ Y_n= & {} a \oplus \beta \otimes \tilde{X} + \epsilon ,\nonumber \\ r= & {} (p \le \alpha \rightarrow r) \wedge (p \ge \alpha \rightarrow t) \end{aligned}$$

where *C* represents a closing operation in the sub-composition and $p=P(\beta = 0)$ is the probability of the slope being 0.

The compositional fold-change (cFC) reported by REPAC represents the fold-change of the isometric log-ratio transformed compositions. This effect size metric, must be interpreted in the simplex space. Since interpretation of changes in the simplex space are not easy to interpret, the REPAC package also provides the mean composition changes across groups (mcDiff) which can be interpreted as the mean percentage in the PAS usage compared to the reference site. REPAC also provides pre-annotated intervals of 3′UTR events for both human and mouse based on polyAsite database [14].

### Simulation benchmark

To assess the performance of REPAC, we used simFlux [27] to simulated fastq files of the dataset with 2 conditions and 5 replicates containing 5000 genes with two isoforms: a normal one (annotated) and one which 3′-UTR were extended by 1000 bp from the original gene model. Each replicates contained 5000 non-overlapping gene models that were randomly selected from the GENCODE 37 annotation and were composed of 2500 genes with longer/shorter isoforms equally distributed and 2500 genes without preferential isoform. The overall expression levels of the genes for each replicate were randomly assigned and the proportion of extended isoform usage was randomly assigned proportion *p* ($0.1 \ge p \le 0.35$) in the first condition and $1-p$ for the second condition. The fastq files were aligned with STAR with default parameters against the hg38 human genome. For the benchmark of REPAC, we quantified 50 bp windows located 100 bp upstream of the PAS (TTS of the isoforms) using featureCounts, and the resulting matrix was used as input to REPAC to compare the two simulated conditions. Similarly, for DaPars [6] we generated wig files from the alignments and used them as input to DaPars along with a gtf file of the simulated isoforms. To benchmark QAPA [4], we quantified the 3′-UTR expression with salmon v1.6.0 [10] using an index of 3′-UTR sequences extracted with the QAPA helper script and passed the quantification as input to QAPA. Finally, we assessed LABRAT [9] performance by extracting the last two exons of the simulated transcripts using the mode “makeTFfasta” and quantified it using the modes “runSalmon” and “calculatepsi.” The absolute effect size of each method, i.e., cFC, $\Delta PDUI$, $\Delta PAU$, and $\psi$ for REPAC, Dapars, QAPA, and LABRAT, respectively, was used as a score variable to compute the empirical ROC curves and AUC with the R/Bioconductor package ROCit.

### Resources and pre-processing

Both REPAC and QAPA rely on pre-annotated PAS to infer DPU. In this work, we used PAS annotations from the PolyAsite database for both mouse (mm10) and human (hg38) genomes. For each genome, we processed the PAS using *QAPA build* to incorporate PolyASite into the latest Ensembl annotations for each genome (v102 and v105 for mm10 and hg38, respectively) and select PAS overlapping with 3′-UTR regions. A total of 67509 and 85476 PAS coordinates were obtained and used in the downstream analysis for mice and humans, respectively. For the QAPA analysis, we extracted the 3′-UTR sequences using *QAPA* fasta. These sequences were used to build the index and perform the 3′-UTR expression quantification with salmon [10]. For the REPAC analysis, we generated a BED file with coordinates for 50 bp windows located 50 bp upstream of the PAS (herein referred to just as polyadenylation sites), which were used as input to the REPAC package. The whole transcriptome index for salmon [10] was built from the latest set of CDS and ncRNAs for the mouse genome from the Ensembl website to obtain the gene level quantification (see the “Technical implications of APA in downstream analyses” section).

### Differential polyadenylation in activated B cells

Differential polyadenylation usage analyses were performed using REPAC and QAPA (v1.3) [4] to detect differential PAS usage between naive and activated B cells. The REPAC analysis was carried out using the REPAC package to query recount3 tracks and quantify PAS to obtain an expression matrix. Next, we filtered low expressed sites ($$CPM < 10$$) and low expressed genes ($$CPM < 30)$$ from the analysis using the salmon whole transcriptome quantification (see the “Technical implications of APA in downstream analyses” section) using the approach described in Chen and collaborators [28]. Finally, we estimated the cFC between PAS by fitting a linear model on the ilr-transformed compositions. Compositions with $$|cFC| \ge 0.25$$ and adjusted *p*-value $$\le 0.05$$ were considered shortening or lengthening events if their cFC were negative or positive, respectively. For the GSEA analysis, we ranked the results of REPAC by *t*-statistics and tested the MSigDB GO Biological process collection for negative enrichment using a Monte Carlo adaptive multilevel splitting approach, implemented in the fgsea package [29]. The results of GSEA were collapsed with fgsea collapsePathways function to reduce redundancy.

For the QAPA analysis, we used the QAPA quant option to load 3′-UTR expression estimates from salmon and compute the PAU’s for each sample. Low expressed 3′-UTRs were filtered (CPM $\le$ 10), and the $\Delta PPAU$ was computed as the difference between the average PPAU for each condition. Finally, genes with $|\Delta PPAU| \ge 20$ were considered shortening or lengthening events if their $\Delta PPAU$ were negative or positive, respectively. Similarly, for the LABRAT analysis we generated the indexes using the mode “makeTFfasta” with the GENCODE M30 annotations, quantified it using the mode “runSalmon,” and computed the psi values and *p*-values with the “calculatepsi” mode. Enrichment analysis of GO BP were conducted in the same manner as REPAC, but instead ranking the genes by $\Delta PPAU$ and *psi* for QAPA and LABRAT, respectively.

### Technical implications of APA in downstream analyses

To assess the impact 3′-US events can have in gene expression estimations, we obtained the raw data from SRA (SRP048707 [13]) and estimated the transcripts expression levels with salmon [10]. The transcripts estimates were summarized at gene level with tximport [26] setting the argument “countsFromAbundance” to “no” (uncorrected expression values) and “lengthScaledTPM” (isoform length-corrected values). To evaluate whether the differences, if any, observed after length correction were a product of alternative splicing, we also performed the same gene expression estimations using only coding sequences (CDS). For each estimate, low count genes (< 10 CPM) were filtered and the remaining genes were normalized with the trimmed mean of the *M*-Values method. A generalized linear model approach coupled with empirical Bayes moderation of standard errors and voom precision weights [30, 31] was used to detect deferentially expressed genes between the selected contrasts. Adjusted *p*-values controlling for multiple hypothesis testing were performed using the Benjamini-Hochberg method [32]. Next, the ratio between the fold-change of the two results for the genes with significant DPU was used to estimate the impact on downstream analysis.

### Analysis of GTEx tissues

To evaluate how well REPAC can scale with large data sets, we randomly selected a subset of 20, 100, 200, and 400 brains (cortex) and testis samples from the GTEx tracks of recount3 [8]. For each subset, we quantified the PAS using the REPAC function *create_pa_rse* and recorded the time taken to quantify each subset. Next, we filtered PASs that were lowly expressed (counts $\le$ 10) and estimated the cFC between PAS by fitting a linear model on the ilr-transformed compositions. PAS with a $|cFC| \ge 0.25$ and $FDR \le 0.05$ were considered shortening or lengthening events if their cFC were negative or positive, respectively. GSEA analysis was carried out by ranking the results of REPAC by *t*-statistics and testing the MSigDB hallmarks collection with the fgsea package [29].

## Supplementary Information

**Additional file 1:** **Supplementary Figures.**

**Additional file 2:** **Supplementary Tables.**

**Additional file 3.** Peer review history.

## Data Availability

All raw data used in this work are available at the Sequence Read Archive (SRA) (https://www.ncbi.nlm.nih.gov/sra) study SRP048707. Counts estimates from GTEx are available through the recount3 and REPAC R packages. The REPAC R package can be obtained at https://github.com/eddieimada/REPAC [33]. All scripts to reproduce the analyses in this manuscript, including simulation, are available at https://github.com/eddieimada/REPAC_paper [34] and Zenodo [35].
