# Supplementary material for: REPAC: analysis of alternative polyadenylation from RNA-sequencing data
Source: Genome Biol. 2023 Feb 9;24:22. doi: 10.1186/s13059-023-02865-5 (PMC9912678; doi:10.1186/s13059-023-02865-5)
Supplement: Supplementary file 1 — Additional file 1: Supplementary Figures. [file 13059_2023_2865_MOESM1_ESM.pdf]

Supplementary Figures

Table of Contents

Figure S1 – Simulation Benchmarks **..2**

Figure S2 – Brain vs Testis analysis **..3**

Figure S3 – Top 10 results for QAPA **..4**

Figure S4 – Top 10 results for LABRAT **..5**

Figure S5 – Top 10 results for REPAC **..6**

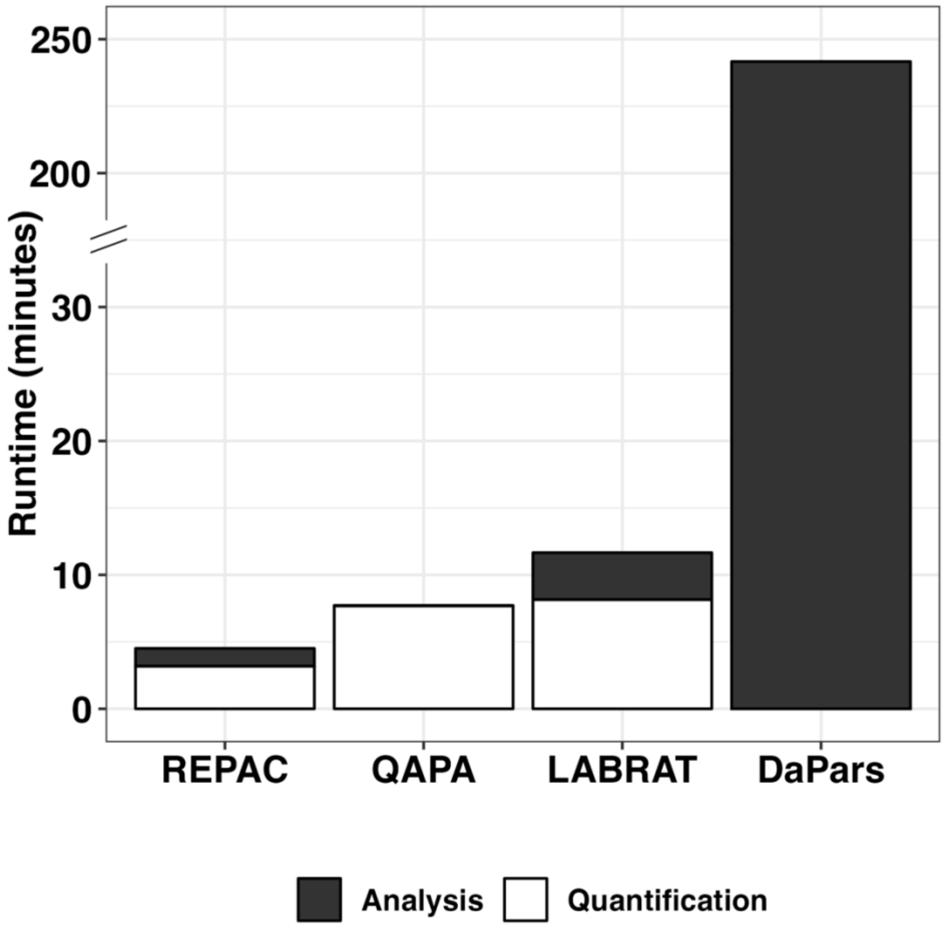

Figure S1 - Simulation runtime between each method benchmarked.

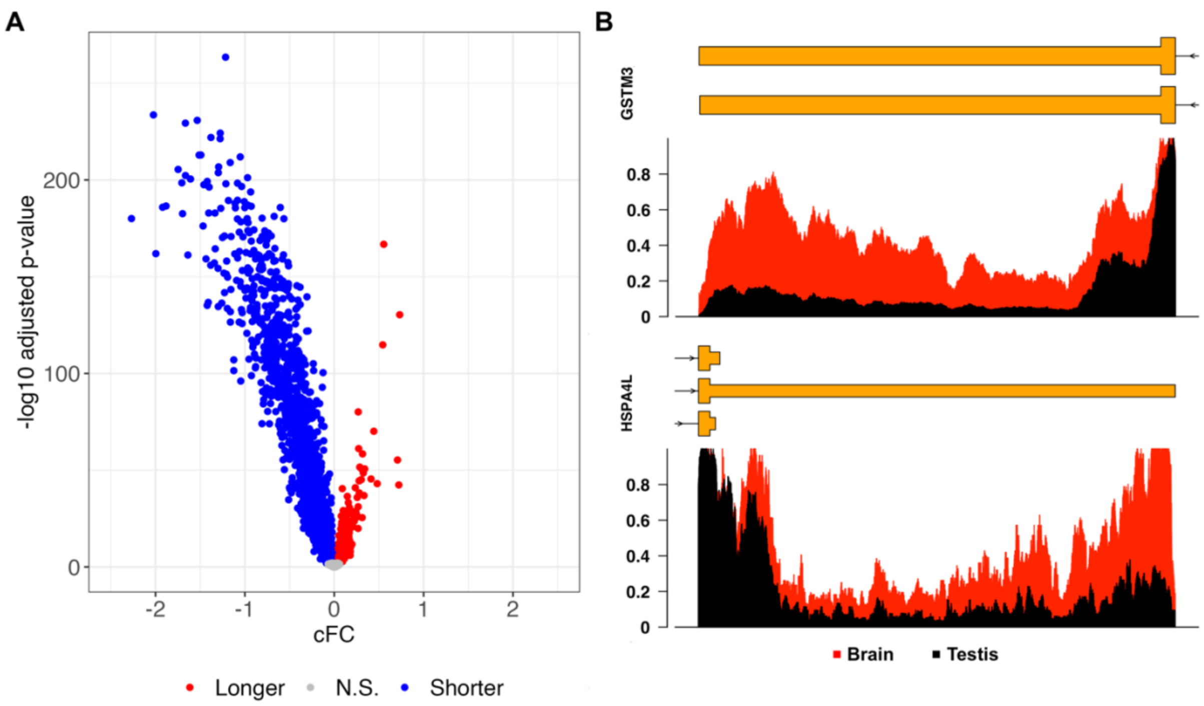

Figure S2 - GTEx comparison of brain vs testis tissues. A) Volcano plot from REPAC results shows that shorter 3'-UTR isoforms are used in testis compared to brain tissues. B) 3'-UTR normalized coverage of genes with alternative polyadenylation between brain and testis detect by REPAC.

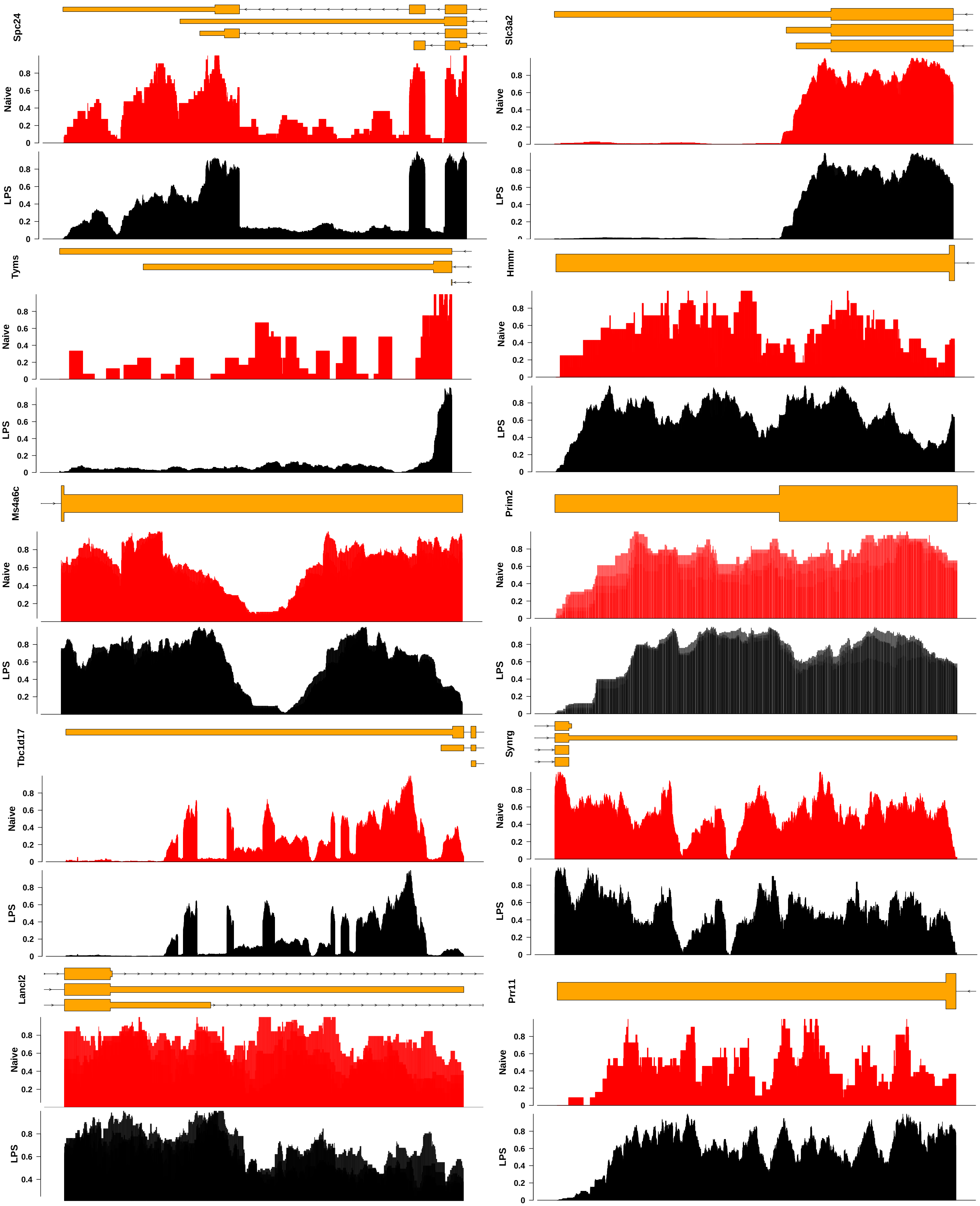

Figure S3 - 3'-UTR coverage across Naive and LPS-activated B cells for the top 10 APA events detected by QAPA.

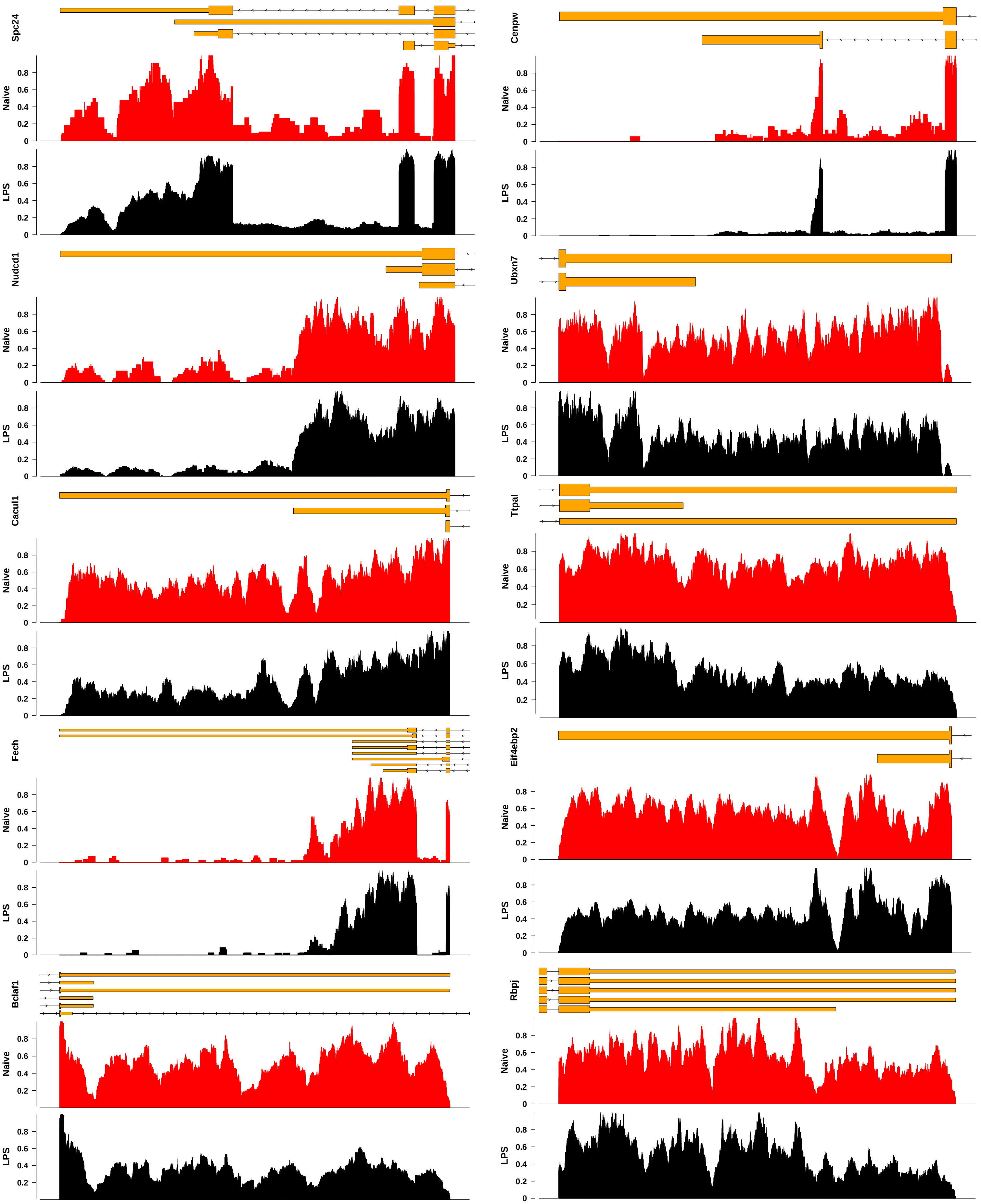

Figure S4 - 3'-UTR coverage across Naive and LPS-activated B cells for the top 10 APA events detected by LABRAT.

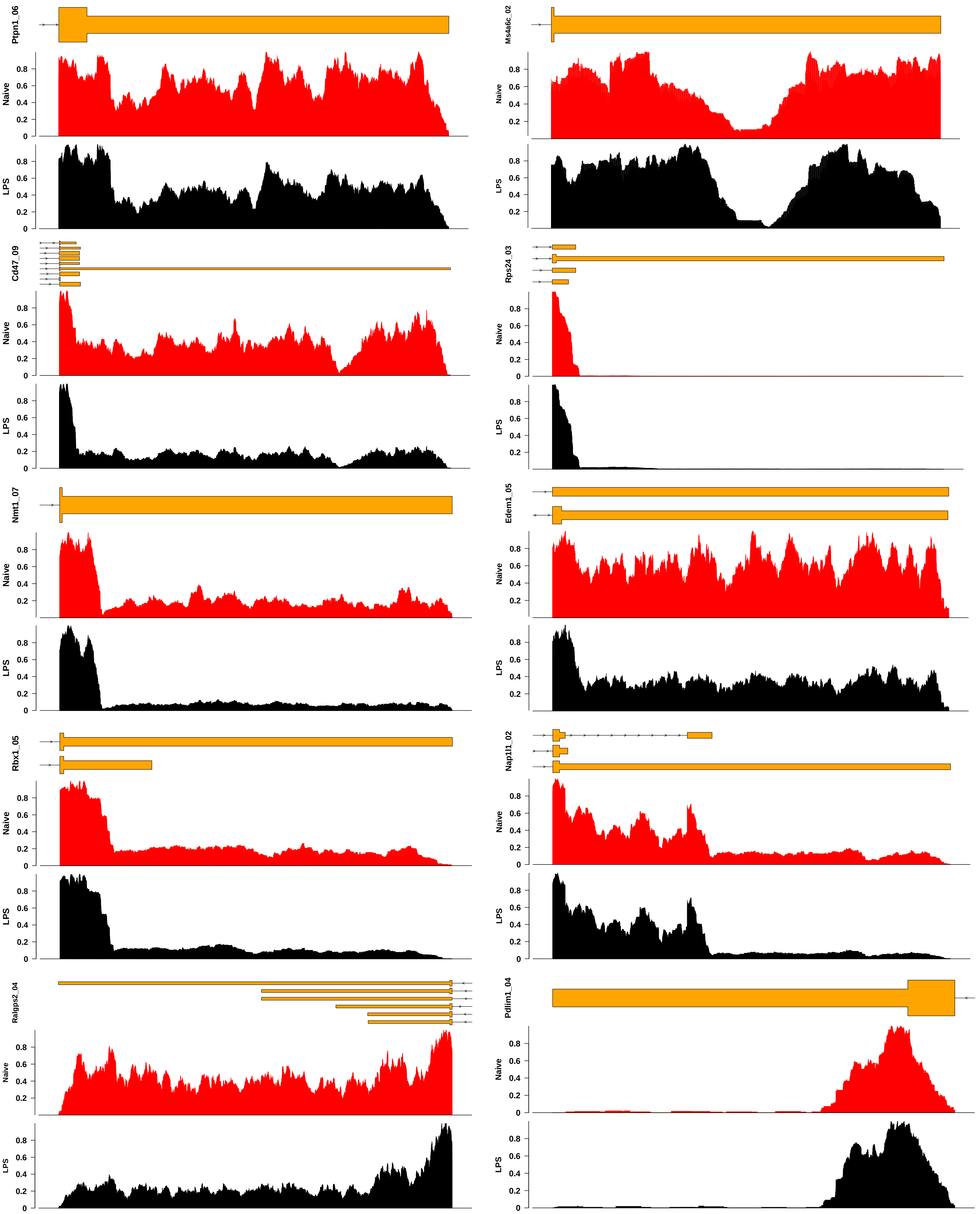

Figure S5 - 3'-UTR coverage across Naive and LPS-activated B cells for the top 10 APA events detected by REPAC.
