# Supplementary material for: REPAC: analysis of alternative polyadenylation from RNA-sequencing data
Source: Genome Biol. 2023 Feb 9;24:22. doi: 10.1186/s13059-023-02865-5 (PMC9912678; doi:10.1186/s13059-023-02865-5)
Supplement: Supplementary file 3 — Additional file 3. Peer review history. [file 13059_2023_2865_MOESM3_ESM.docx]

**Review history**

**First round of review**

**Reviewer 1**

This manuscript by Imada et al. reports a new method, named REPAC, for APA analysis using RNA-Seq data. The major novelty of this method is its speed, which is due to its use of recount3 data. Using simulated as well as real data, the authors show that REPAC is faster than two other commonly used methods, DaPars and QAPA. The performance of REPAC, as measured by AUC, is also higher than those of the two other methods. The authors also found that genes in some key biological processes are particularly subject to APA regulation during B cell activation by LPS. Overall, the strength of their method in speed appears quite convincing, other technical advantages are not clear.

Major:
* The authors claim that REPAC can handle complex comparisons, i.e., factorial designs, paired samples, etc. They need to provide data to support this claim.
* It appears that REPAC does not handle intronic polyadenylation cases, which are widespread in the genome. The authors are recommended to incorporate this function, so that their method could be more complete for all types of APA events.
* The correlation between APA and gene expression regulation in B cell activation is interesting. But the authors need to avoid using the same reads for both analyses, which could lead to compounding issues. I suggest the authors use CDS reads only for gene expression.

Minor:
* Page3, line 17, "DaPars was drastically slower (> 40-fold) than REPAC and QAPA". The authors need to show more details in figures/tables.
* Page4, line 13, Figure 1B should be Figure 2B?
* Page4, line 14, the results described in this paragraph should be supported by figures/tables.
* Page4, line 54, the authors compared REPAC with QAPA using delta cFC vs delta PPAU. The cutoff for cFC is 0.25, while PPAU is 20. Are these two cutoffs comparable?
* Page5, line 33, Figure 4B, it is not clear what statistical significance cutoff was used to determine gene expression level changes.

**Reviewer 2**

In this manuscript, Imada and colleagues describe a new computational method for quantifying alternative polyadenylation (APA) in RNAseq datasets. This method, termed REPAC, is able to efficiently query many publicly available datasets through the use of recount3, making it very useful for large scale analyses that use many samples. Although the approach is unique in its ability to allow the analysis of many samples, the manuscript as presented has important deficiencies. These include a lack of testing of REPAC against RNAseq datasets of varying parameters and qualities, insufficient evidence that it performs better than an existing tool, QAPA, with regards to performance on real data, and a lack of understanding how the biology behind APA may affect the ability of REPAC to accurately report on the quantification of specific APA events. These concerns are laid out in detail below.

MAJOR COMMENTS

1. In figure 2, the authors benchmark REPAC and two other APA quantification tools, QAPA and DaPars using a simulated RNAseq dataset. The authors find that QAPA and REPAC both perform quite well. This is certainly encouraging. However, the main advantage of REPAC is that it is able to query many, many datasets. These publicly available datasets will no doubt vary greatly in their parameters (read depth, read length, single/paired end, RNAseq vs 3' end libraries, etc.) It is difficult to judge how REPAC will be able to handle such variations when only a single simulated dataset is presented. The authors could mitigate this weakness by repeating the simulation using varied read depths, lengths, etc. It might be predicted that since REPAC only uses read depth in a defined 50 bp window for each APA event for its calculations, it could be more sensitive to lower depth than other methods that use read counts across a longer transcript region.

2. Related to this, how well does REPAC do with APA sites that are quite close to each other? If REPAC is counting reads in a 50 bp window that is 100 bp upstream of the polyA site, what happens if there are polyA sites that are less than 100 bp apart? In those cases, the sequence that is unique to the downstream site would not be queried, likely lowering accuracy. Although these events are rare, it is another weakness of simply counting reads in a defined window, especially if the parameters for that window (length, position, etc.) are constant and do not vary according to the topology of the gene's APA sites.

3. Does the read counting of these windows take strand into account? Does recount3 know whether a given library was stranded or not? This is important because there are many cases where 3' UTRs of neighboring genes that are in "tail to tail" orientation overlap but on different strands. Having stranded counts would allow these genes to be differentiated.

4. There are a few different flavors of APA, namely cases where multiple polyadenylation sites are present within the same exon (sometimes called tandem UTRs), cases where to different polyadenylation sites lie in different, often mutually exclusive exons (sometimes called alternative last exons), and cases where cryptic intronic polyA sites that are normally ignored are used (sometimes called intronic APA). How well REPAC handles each of these event types is unknown. All of the plots for individual genes that are shown are of the tandem UTR variety.

5. The metric that REPAC reports for APA, abs(cFC) is not defined anywhere. This makes it very difficult to understand what this metric means and how to interpret it. Further, when the authors set thresholds for analyses (e.g. abs(cFC) >= 0.25), it makes it difficult to interpret these data.

6. The authors analyze data from naive and activated B cells using QAPA and REPAC. They conclude that REPAC performed better with this data. This may certainly be true, but at least part of the justification presented is that a GSEA analysis of the REPAC results yielded terms related to B cell biology while a GSEA analysis of the QAPA results did not. This is not nearly enough evidence to back up the claim that REPAC performs better with real world data. In general, GSEAs are weak evidence for many statements in biology and are not an appropriate metric for benchmarking computational methods. I do sympathize with the authors, though, as it can be difficult to make straightforward comparisons with real data when the ground truth is not known. Perhaps experimental validations (northern blots, RT-qPCR, etc.) of events that REPAC detects but QAPA does not could help. However, I realize that as the authors are primarily computational biologists, this may not be feasible.

7. It's unclear how the results might change if some of the thresholds for this comparison (e.g. delta PPAU >=20, abs(cFC) >= 0.25) were changed. Perhaps these are too restrictive.

8. The authors mention that a potential reason for the inferior performance of QAPA on the real data may be the fact that (1) QAPA does not perform statistical tests to identify significantly regulated genes and (2) most of the genes QAPA did identify were likely false positives due to low expression. To test this, the authors could consider including LABRAT (Goering et al BMC Genomics 2021) in this analysis. LABRAT and QAPA use very similar lightweight alignment approaches, but LABRAT incorporates a test to identify significantly regulated genes and also incorporates an expression filter to exclude lowly expressed genes.

9. Somewhat confusingly, although the authors present the inclusion of lowly expressed genes in QAPA results as a potential reason for its poor performance yet state in the methods that such genes (TPM <= 5) were filtered out of the analysis.

10. In figure 3b, the authors present evidence of a connection between APA usage and gene expression for 14 genes. Such a connection has been observed repeatedly before (for example, PMID 19703394 and PMID 34174817 among others), but this is not referenced here.

11. In figure 3b, what are the units for the colorbar?

12. In figure 3a, what are the units for the y-axis?

13. The authors say that "our results indicated that APA events in 3'-UTR regions can drastically impact gene size". I understand what the authors are trying to say, but this is a problematic comment. First, the size of transcripts change due to APA. The size of the gene remains unchanged. Second, APA events almost by definition change transcript size. This is not a new result obtained by running REPAC.

MINOR COMMENTS

1. Some figures, for example 4A, are blurry to the point of their text being unreadable.

**Authors’ response to reviewers**

We would like to thank the editor and the reviewers for their very helpful and constructive suggestions and for their overall positive comments on our manuscript entitled “Unleashing alternative polyadenylation analysis with REPAC”. We have therefore revised our analyses to address the points raised by the reviewers and streamlined the manuscript to better convey and reflect these changes in the manuscript. Below we summarize these changes and include our answers to the reviewers’ comments.

Comments from Reviewer #1:

This manuscript by Imada et al. reports a new method, named REPAC, for APA analysis using RNA-Seq data. The major novelty of this method is its speed, which is due to its use of recount3 data. Using simulated as well as real data, the authors show that REPAC is faster than two other commonly used methods, DaPars and QAPA. The performance of REPAC, as measured by AUC, is also higher than those of the two other methods. The authors also found that genes in some key biological processes are particularly subject to APA regulation during B cell activation by LPS. Overall, the strength of their method in speed appears quite convincing, other technical advantages are not clear.
Response: We thank the reviewer for the positive comments on our manuscript. We hope to have addressed the reviewer concerns over the technical advantages of REPAC bellow.

Major comments

1. The authors claim that REPAC can handle complex comparisons, i.e., factorial designs, paired samples, etc. They need to provide data to support this claim.
Response: We appreciate the reviewer suggestion to improve our manuscript.
We would like to supply additional explanation to the reviewer that one of the key advantages of REPAC is using the isometric log-ratio transformation which makes compositional data follow a Gaussian distribution. This transformation remove statistical constrains of compositional data allowing us to apply classical methods developed for this type of distribution. Our method uses generalized linear models in the same fashion as the popular R/Bioconductor limma package which supports the complex comparisons mentioned in the manuscript.
To show that REPAC can indeed handle these comparisons, we have addressed the reviewer comment by simulating a factorial/interaction design experiment (see section 9.5 in limma user guide). Briefly, we simulated 4 groups A, B, C, and D were the differences between the differences of B-A and D-C ((B-A) – (D-C)) were expected to exhibit shortening, lengthening, and no preference. We show that REPAC still maintains a high accuracy of 0.95 in this simulation (additional file 2).

2. It appears that REPAC does not handle intronic polyadenylation cases, which are widespread in the genome. The authors are recommended to incorporate this function, so that their method could be more complete for all types of APA events.
Response: We thank the reviewer for the suggestion, and we apologize for the confusion. We would like to clarify that REPAC currently can handle intronic polyadenylation (IPA) cases. REPAC requires annotated polyadenylation sites (PAS) as input and while we do not provide pre-annotated intronic PAS, the user is still able to provide intronic sites if he/she wishes.
The decision to not provide intronic PAS in the package before our first submission was made after much deliberation. IPA events are much more challenging to accurately identify. For instance, we need to make sure that the coverage observed in the intronic region is not a product of alternative splicing events such as alternative donor site or intron retention. Moreover, in certain protocols (i.e., ribosome depletion) the sequencing of unspliced transcripts is very common, which leads to significant noise across all intronic regions. These facts make the identification of IPA not a trivial task. While we completely agree with the reviewer(s) that IPA events are a type of event of great interest, we are afraid that implementing a solution that overcomes the challenges above would not be feasible in a timely manner, but it is something we are actively working on for the next iteration of REPAC. We would also like to note that to the best of our knowledge, currently there is no software that currently supports IPA detection while addressing the challenges mentioned above.

3. The correlation between APA and gene expression regulation in B cell activation is interesting. But the authors need to avoid using the same reads for both analyses, which could lead to compounding issues. I suggest the authors use CDS reads only for gene expression.
Response: We appreciate the reviewer suggestion to improve our analysis. We would like to clarify that the quantifications for APA and gene expression did not originate from the procedure. APA quantifications were extracted from recount3 tracks (which were originally aligned with STAR) and gene expression quantification was perform through quasi-mapping using salmon. Our understanding of the reviewer suggestion was to try to discern whether the effect observed after correction were due 3’UTR length, or due changes in the CDS (alternative splicing). To this end we repeated the quantifications using only the CDS region of the genes and updated the figure to include gene expression estimates of CDS region only. The new figure shows that length correction had little to no effect on the expression estimates of CDSs, indicating that the differences observed in the whole transcripts quantifications after length correction were not caused by changes in the CDS region.

Minor comments

4. Page3, line 17, "DaPars was drastically slower (> 40-fold) than REPAC and QAPA". The authors need to show more details in figures/tables.
Response: We have added a supplementary figure with details of the simulation analysis runtime.

5. Page4, line 13, Figure 1B should be Figure 2B?
Response: We apologize and thank the reviewer for spotting our mistake. We have edited the manuscript to refer to the correct figure.

6. Page4, line 14, the results described in this paragraph should be supported by figures/tables.
Response: We have added a supplementary figure with the results of this analysis and a few examples (see additional file 3).

7. Page4, line 54, the authors compared REPAC with QAPA using delta cFC vs delta PPAU. The cutoff for cFC is 0.25, while PPAU is 20. Are these two cutoffs comparable?
Response: Each tool estimates the effect size using different approaches; therefore, the effect sizes of all tools are not directly comparable. For the accuracy estimation, we used the cutoffs that were used in the original publication, but we also tested other cutoffs to make sure the cutoffs were appropriate and we chose the ones that yielded the highest accuracy in the simulations (which were the ones used in the original publications, hence why we did not report this as a result). We would like to note that the AUC is estimated across all estimated effect sizes without setting a cutoff, which provide an accurate performance estimation independent of the cutoff used.

8. Page5, line 33, Figure 4B, it is not clear what statistical significance cutoff was used to determine gene expression level changes.
Response: We have added the statistical cutoff used to determine differential gene expression in the manuscript (|logFC| ≥ 0.5 and adjusted p-value ≤ 0.05).


Comments from Reviewer #2:

In this manuscript, Imada and colleagues describe a new computational method for quantifying alternative polyadenylation (APA) in RNAseq datasets. This method, termed REPAC, is able to efficiently query many publicly available datasets through the use of recount3, making it very useful for large scale analyses that use many samples. Although the approach is unique in its ability to allow the analysis of many samples, the manuscript as presented has important deficiencies. These include a lack of testing of REPAC against RNAseq datasets of varying parameters and qualities, insufficient evidence that it performs better than an existing tool, QAPA, with regards to performance on real data, and a lack of understanding how the biology behind APA may affect the ability of REPAC to accurately report on the quantification of specific APA events. These concerns are laid out in detail below.
Response: We thank the reviewer for the constructive feedback on our manuscript and we are glad that he/she/them found our new method very useful for large scale analyses. We hope to have addressed the reviewer concerns on some of our analyses.

Major comments

1. In figure 2, the authors benchmark REPAC and two other APA quantification tools, QAPA and DaPars using a simulated RNAseq dataset. The authors find that QAPA and REPAC both perform quite well. This is certainly encouraging. However, the main advantage of REPAC is that it is able to query many, many datasets. These publicly available datasets will no doubt vary greatly in their parameters (read depth, read length, single/paired end, RNAseq vs 3' end libraries, etc.) It is difficult to judge how REPAC will be able to handle such variations when only a single simulated dataset is presented. The authors could mitigate this weakness by repeating the simulation using varied read depths, lengths, etc. It might be predicted that since REPAC only uses read depth in a defined 50 bp window for each APA event for its calculations, it could be more sensitive to lower depth than other methods that use read counts across a longer transcript region.
Response: We thank the reviewer for the suggestion to improve our manuscript. We have performed 4 additional simulations with shorter reads, polyA capture (3’end), single-ended library, and an interaction term suggestion by reviewer #1. The results of these new simulations are included in the manuscript and in a new supplementary table (see additional file 2). Overall, REPAC still maintained a high accuracy (>0.95) across all simulated conditions.

2. Related to this, how well does REPAC do with APA sites that are quite close to each other? If REPAC is counting reads in a 50 bp window that is 100 bp upstream of the polyA site, what happens if there are polyA sites that are less than 100 bp apart? In those cases, the sequence that is unique to the downstream site would not be queried, likely lowering accuracy. Although these events are rare, it is another weakness of simply counting reads in a defined window, especially if the parameters for that window (length, position, etc.) are constant and do not vary according to the topology of the gene's APA sites.
Response: The function in the REPAC package used to quantify the polyA sites (PAS) requires a GRanges object from the R/Bioconductor package GenomicRanges as input. This object must contain the intervals upstream of the PAS that will be quantified. While in the package we provide a pre annotated collection of 50bp intervals, this function can receive intervals of any length, which can be defined by the user. Because REPAC has separate functions to quantify the sites and to perform the statistical tests, REPAC can also be used with alternative workflows; For instance, any quantification software (e.g., featureCounts, HTseq, etc.) can be used to quantify the counts in the intervals if the user wishes to start from their own bam files instead of recount3 quantifications (this is how the simulation quantifications were done). Likewise, the user can choose to quantify the 3’UTR using salmon similarly to QAPA or LABRAT if he/she/they wishes. As long the user is able to generate quantifications for each PAS and structure it in the required format, REPAC offers a great degree of flexibility on how the user can quantify the PAS to perform the regression of compositions.

Regarding how REPAC can deal with close PASs, ultimately, it is up to the user to evaluate if such close PASs are biologically relevant or should be remove due redundancy. REPAC can still perform the pairwise comparisons even if the PASs are in close proximity, in the worst case scenario, keeping such sites will at most slightly impact the computing time.

We appreciate the observation made by the reviewer and we have extended the discussion section in the manuscript and in the user guide to make it clearer to the user/reader that quantification of PAS can be performed through other venues other than the supplied pre-annotated PAS intervals.

3. Does the read counting of these windows take strand into account? Does recount3 know whether a given library was stranded or not? This is important because there are many cases where 3' UTRs of neighboring genes that are in "tail to tail" orientation overlap but on different strands. Having stranded counts would allow these genes to be differentiated.
Response: We agree with the reviewer that stranded quantification is important due to the fact mentioned above. We would like to clarify that recount3 harbors data for over 750,000 samples, which as one can imagine, it takes a considerable amount of storage to keep this data available for users to query. This data is stored in a bigwig format, which does not support strandness, unless generating two set of files for each strand. This would double the already huge storage requirements of recount3.

Nevertheless, while quantifications pulled from recount3 are not stranded, the user can still use the methods mentioned in the comment #2 to perform stranded quantification of PASs from a bam file. Alternatively, the user can also opt to quantify the nearest non-overlapping region as a proxy to a redundant site.

We have a new function that allows the user to quantify the PAS directly from bam files and set their own parameters (strandness, unique mapping, etc.). We also edited the manuscript to make this fact clearer and updated the user guide on how to proceed if they wish to process stranded libraries.

4. There are a few different flavors of APA, namely cases where multiple polyadenylation sites are present within the same exon (sometimes called tandem UTRs), cases where to different polyadenylation sites lie in different, often mutually exclusive exons (sometimes called alternative last exons), and cases where cryptic intronic polyA sites that are normally ignored are used (sometimes called intronic APA). How well REPAC handles each of these event types is unknown. All of the plots for individual genes that are shown are of the tandem UTR variety.
Response: We agree with the reviewer that other flavors of APA are of interest, as this was also noted by reviewer #1. We would like to clarify that while REPAC currently can handle intronic polyadenylation (IPA) and alternative exons cases, however we opted to not include pre-annotated PAS that are not of the tandem UTR variety in the package.

The decision to not provide these flavors of APA in the package before our first submission was made after much deliberation. Alternative last exons (ALE) often co-occur with alternative splicing (AS) which makes it very challenging to discern if the phenotype observed is a product of APA, AS, or both. IPA events are much more challenging to accurately identify. For instance, we need to make sure that the coverage observed in the intronic region is not a product of AS events such as alternative donor site or intron retention. Moreover, in certain protocols (i.e., ribosome depletion) the sequencing of unspliced transcripts is very common, which leads to significant noise across all intronic regions. These facts make the identification of IPA not a trivial task.

While we completely agree with the reviewer(s) that other flavors of APA are of great interest, we opted to not encourage the analysis of these sites for now (experienced researchers that are aware of these caveats can still analyze them) while we are working on developing a solution that overcomes the challenges above in a manner that is friendly to the user.

We would like to note that we are actively working on this feature for the next iteration of REPAC. We would also like to note that to the best of our knowledge, currently there is no software that currently supports the detection of IPA/ALE while addressing the caveats mentioned above.

5. The metric that REPAC reports for APA, abs(cFC) is not defined anywhere. This makes it very difficult to understand what this metric means and how to interpret it. Further, when the authors set thresholds for analyses (e.g. abs(cFC) >= 0.25), it makes it difficult to interpret these data.
Response: We have added a description of the effect size estimate cFC in the methods section to clarify how to interpret it. cFC stands for compositional fold-change and it represents the fold-change between the compositions in the Simplex space (equation 1, see Aitchison J., 1982) or in other words it is the slope (β, equation 3) of the generalized linear model fitted for the compositions after the isometric log-ratio transformation. Since changes in the Simplex space can be hard to directly interpret, we have updated the function to also output a value “mcDiff” which stands for “mean composition differences” and represents the changes between conditions in the Euclidean space (changes in % of PAS usage). This new value is also described in the manuscript.

6. The authors analyze data from naive and activated B cells using QAPA and REPAC. They conclude that REPAC performed better with this data. This may certainly be true, but at least part of the justification presented is that a GSEA analysis of the REPAC results yielded terms related to B cell biology while a GSEA analysis of the QAPA results did not. This is not nearly enough evidence to back up the claim that REPAC performs better with real world data. In general, GSEAs are weak evidence for many statements in biology and are not an appropriate metric for benchmarking computational methods. I do sympathize with the authors, though, as it can be difficult to make straightforward comparisons with real data when the ground truth is not known. Perhaps experimental validations (northern blots, RT-qPCR, etc.) of events that REPAC detects but QAPA does not could help. However, I realize that as the authors are primarily computational biologists, this may not be feasible.
Response: We are thankful to the reviewer for sympathizing with the fact that our group is computational, and we are afraid experimental validation would not be currently feasible in timely manner. In addition, we completely agree with the reviewer comment that GSEA alone is weak evidence for biological discovery. We apologize if the way we phrase these results were not clear, we have updated the discussion to clarify the following rationale: The goal of our B Cell activation study was not to claim that the 3’UTR shortening of the secretion pathway genes was a novel discovery. We choose this model for a case study specifically the APA events occurring in B Cell transition to plasm cell has been recently described*. APA was first discovered in B cells and a recent manuscript* provided extensive evidence that secretion pathway genes have shorter 3’UTR in plasma cells (LPS-activated B cells). Therefore, our rationale was that these findings could be used as a “positive control” to evaluate if the methods can achieve the same finding that has already been confirmed by others.
Nevertheless, we also agree that failing to recover known B cell biology does not necessarily generalize that the tools would fail in other real data scenarios, and we have updated the language in the discussion to avoid claiming that REPAC performs better in real data, but instead, that it was able to recover more information in this particular case.

*Cheng LC, Zheng D, Baljinnyam E, Sun F, Ogami K, Yeung PL, et al. Widespread transcript shortening through alternative polyadenylation in secretory cell differentiation. Nature communications. 2020;11(1):1–14.

7. It's unclear how the results might change if some of the thresholds for this comparison (e.g. delta PPAU >=20, abs(cFC) >= 0.25) were changed. Perhaps these are too restrictive.
Response: For the accuracy estimation, we used the cutoffs that were used in the original publication, but we also tested other cutoffs to make sure the cutoffs were appropriate and we chose the ones that yielded the highest accuracy in the simulations (which were the ones used in the original publications, hence why we did not report this as a result). We would like to note that the AUC is estimated across all estimated effect sizes without setting a cutoff, which provide an accurate performance estimation independent of the cutoff used.


8. The authors mention that a potential reason for the inferior performance of QAPA on the real data may be the fact that (1) QAPA does not perform statistical tests to identify significantly regulated genes and (2) most of the genes QAPA did identify were likely false positives due to low expression. To test this, the authors could consider including LABRAT (Goering et al BMC Genomics 2021) in this analysis. LABRAT and QAPA use very similar lightweight alignment approaches, but LABRAT incorporates a test to identify significantly regulated genes and also incorporates an expression filter to exclude lowly expressed genes.
Response: We thank the reviewer for the suggestion as we were not aware of the LABRAT software. We have included results for LABRAT in the simulation benchmark and for the B cell study.

As pointed by the reviewer, the methods use very similar approaches, hence we found that in the simulation LABRAT perform well (similar to REPAC and QAPA). For the B cell study, LABRAT also failed to recover APA events in genes associated with secretion pathway and did not show enrichment in IRE1 response.

9. Somewhat confusingly, although the authors present the inclusion of lowly expressed genes in QAPA results as a potential reason for its poor performance yet state in the methods that such genes (TPM <= 5) were filtered out of the analysis.
Response: We apologize for the confusion. The filtering approach used was the one implemented in the filterByExpr function of the edgeR R/Bioconductor package which uses the approach by Chen et al*. This approach evaluates expression between groups separately and keeps genes that the expression level passes the threshold in at least one of the groups. Therefore, when we mention lowly expressed genes, we are referring to genes in which one of the groups exhibit high expression, but the other do not. We edited the manuscript to clarify this. Moreover, in order to reduce confusion, we decided to apply the same threshold for REPAC and QAPA (> 10 CPM; the default setting for the function mentioned above). This change did not significantly impact the results or conclusions, other than dropping one gene (Atf3) from Additional File 5.

We would also like to note that this was not the only factor impacting the performance of QAPA since it also captured genes without significant changes in the 3’UTR that were expressed in both conditions.

*Chen, Y., Lun, A. T., & Smyth, G. K. (2016). From reads to genes to pathways: differential expression analysis of RNA-Seq experiments using Rsubread and the edgeR quasi-likelihood pipeline. F1000Research, 5.

10. In figure 3b, the authors present evidence of a connection between APA usage and gene expression for 14 genes. Such a connection has been observed repeatedly before (for example, PMID 19703394 and PMID 34174817 among others), but this is not referenced here.
Response: We thank the reviewer for pointing these manuscripts. We have now referenced them.

11. In figure 3b, what are the units for the colorbar?
Response: The units are standardized CPM (Z-score). We have added a label to the figure to make this clearer.

12. In figure 3a, what are the units for the y-axis?
Response: The y-axis represents the coverage normalized to the highest expressing base, meaning the coverage of each base is divided by the highest value. This provides a unbiased view independent of expression levels as we aim to show differences in the proportion of 3’UTR usage. We have edited the figure legend to include this explanation.

13. The authors say that "our results indicated that APA events in 3'-UTR regions can drastically impact gene size". I understand what the authors are trying to say, but this is a problematic comment. First, the size of transcripts change due to APA. The size of the gene remains unchanged. Second, APA events almost by definition change transcript size. This is not a new result obtained by running REPAC.
Response: We completely agree with the reviewer that transcript size is the appropriate term to be used and have changed accordingly. We also did not intend to claim that these changes were novel results, our intention was to draw attention to the technical implications of this change in transcript size. Therefore, we have edited the text and included the citations provided by the reviewer on comment #10.

Minor comments

14. Some figures, for example 4A, are blurry to the point of their text being unreadable.
Response: We apologize for the inconvenience. We generated figures with a resolution of at least 330ppi, however in our experience as reviewers we find that during the material compilation these figures lose quality. We will further increase the pixels density, however we found that using the link on the top right corner “Click here to access/download” in the figure page allows to obtain the image with the original quality uploaded.

**Second round of review**

**Reviewer 1**

The authors have addressed my concerns.

**Reviewer 2**

The authors have satisfactorily responded to all of my questions and suggestions. I congratulate them on the creation of a useful tool.
